# Ultrasound-guided implantation of radioactive ^125^I seed in radioiodine refractory differentiated thyroid carcinoma

**DOI:** 10.1186/s12885-021-08500-5

**Published:** 2021-07-20

**Authors:** Wei Chen, Yu kun Luo, Ying Zhang, Qing Song, Jie Tang

**Affiliations:** 1 Department of Ultrasound, The Seventh Medical Center, Medical College of PLA, Chinese PLA General Hospital, Beijing, China; 2 Department of Ultrasound, The First Medical Center, Chinese PLA General Hospital, Beijing, China; 3 Department of Ultrasound, The Seventh Medical Center, Chinese PLA General Hospital, Beijing, China

**Keywords:** Differentiated thyroid carcinoma, Lymph node metastasis, Ultrasound, Iodine-125, Brachytherapy

## Abstract

**Background:**

Treatment for radioiodine refractory differentiated thyroid carcinoma (RR-DTC) is challenging. The purpose of this study was to assess the efficacy and safety of ultrasound-guided implantation of radioactive ^125^I-seed in radioiodine refractory differentiated thyroid carcinoma.

**Methods:**

Thirty-six cervical metastatic lymph nodes (CMLNs) diagnosed with RR-DTC from 18 patients were enrolled in this retrospective study. US and contrast-enhanced ultrasound (CEUS) examinations were performed before implantation. Follow-up comprised US, CEUS, thyroglobulin (Tg) level and routine hematology at 1–3, 6, 9 and 12 months and every 6 months thereafter. The volumes of the nodules were compared before implantation and at each follow-up point. The volume reduction rate (VRR) of nodules was also recorded.

**Results:**

The median volume of the nodules was 523 mm^3^ (148, 2010mm^3^) initially, which decreased significantly to 53mm^3^ (0, 286mm^3^) (*P* < 0.01) at the follow-up point of 24 months with a median VRR as 95% (86,100%). During the follow-up period (the range was 24–50 months), 25 (69%) nodules had VRR greater than 90%, of which 12 (33%) nodules had VVR ≈ 100% with unclear structures and only ^125^I seed images were visible in the US. At the last follow-up visit, the serum Tg level decreased from 57.0 (8.6, 114.8) ng/ml to 4.9 (0.7, 50.3) ng/ml, (*P* < 0.01).

**Conclusion:**

US-guided ^125^I seed implantation is safety and efficacy in treating RR- DTC. It could be an effective supplement for the comprehensive treatment of thyroid cancer.

## Background

The world over, cases of thyroid carcinoma have been continuously increasing in recent decades [1–3]. Differentiated thyroid carcinoma (DTC), arising from thyroid follicular epithelial cells, accounts for 90% of these cases [4]. With treatments of surgery, suppression of thyroid stimulating hormone (TSH) and selective treatment of radioactive iodine-131 (RAI-131), the vast majority of DTC patients have an excellent prognosis, as reflected by 5-year relative survival rates of approximately 95% [1, 5]. However, local recurrence and/or distant metastases may occur in 15–30% of patients, and two-thirds of them will become radioiodine refractory (RR) during long-term follow-up [6–10]. RAI-131 therapy is the first-line systemic treatment in postoperative patients with progressive DTC, therefore, treatment options for radioiodine refractory differentiated thyroid carcinoma (RR-DTC) have been hampered, leading to a poor overall prognosis [11]. As an emerging systemic therapy, tyrosine kinase inhibitors (TKIs) have recently shown activity in RR-DTC, however, the lack of long-term survival data and the relatively indolent nature of DTC, it is difficult to determine the appropriate time to initiate TKI therapy [12]. Therefore, localized treatment plays an important role in local recurrence and/or metastases from RR-DTC. Historically, surgery has been the mainstay treatment for locoregional metastases from DTC, however, repetitive neck excision poses a high risk of complications [13, 14]. Therefore, it is desirable to develop alternatives that are less invasive than repeated surgery as part of a multimodal treatment for DTC.

^125^I-seed implantation has been proposed as an excellent treatment for head and neck unmanageable recurrent carcinoma [15–19]. However, the use of this method for the local control of foci from DTC has rarely been reported. In this retrospective study, the efficacy and feasibility of ultrasound (US)-guided ^125^I-seed implantation for cervical metastatic lymph nodes (CMLNs) from RR-DTC were evaluated.

## Methods

### Study subjects

Inclusion criteria: (a) patients were required to be aged≥18 years; (b) patients who underwent thyroidectomy for DTC; (c) patients with measurable, pathological and/or cytologically confirmed CMLN from DTC, and evidence of RR according to at least one criterion used in recent clinical trials as follows (i) at least one lesion that never concentrated iodine-131; (ii) at least one lesion that progressed within 12 months after RAI therapy despite iodine-131 avidity, or persistent disease after a cumulative dose of iodine-131 ≥ 600 mCi; (iii) partial thyroidectomy leading to thyroid tissue residue, which affects the efficacy of RAI-131;and (d) patients who had inoperable CMLN or refused to undergo repeated neck surgical dissection.

The exclusion criteria were as follows: (a) cognitive impairment due to neuropathy or psychosis, (b) severe coagulation disorders; (c) patients with severe heart, respiratory, liver, or renal failures, and (d) pregnant or breastfeeding patients.

We performed a retrospective analysis of 18 patients (11 men and 7 women) with 36 CMLNs who underwent US-guided ^125^I seed implantation in our hospital between June 2016 and November 2018. Of all patients, 17 underwent total thyroidectomy, whereas1 underwent partial thyroidectomy. BRAF^V600E^ mutations were detected in 17 patients. CMLNs were situated at levels I (0 case), II (6 cases), III (12 cases), IV (10 cases), V (1 cases) and VI (7 cases) respectively. The median volume of the initial CMLNs was 523 mm^3^ (148, 2010mm^3^). Three cases exhibited clear symptoms of neck compression due to tumor volumes > 10,000 mm^3^, whereas two cases had dysphagia due to esophageal narrowing under tumor compression. Two patients underwent tracheotomy. One patient had bilateral vocal cord paralysis. One patient had a skin ulceration on the surface of the lesion. Detailed clinical characteristics before implantation of ^125^I-seeds are listed in Table 1.

**Table 1:** Clinical characteristics regarding 18 patients of 36 CMLNs treated with implantation of ^125^I-seeds

Parameter	Characteristics	Result
Sex of patients (*n* = 18)	M/F	11/7^A^
Age of patients (*n* = 18)		52.2 ± 20.8 (27–87) ^B^
Subtypes of DTC (*n* = 18)	PTC/FTC	17/1 (94/6) ^C^
No. of neck surgeries performed (*n* = 18)	< 3/≥3	16/2 (89/11) ^C^
Cumulative dose of RAI-131 (*n* = 18)	< 600/≥600mci	14/4 (78/22) ^C^
BRAF^V600E^ mutation (*n* = 18)	P/N	17/1 (94/6) ^C^
No. of CMLNs (*n* = 18)	1	9 (50) ^C^
	2	3 (17) ^C^
	≥3	6 (33) ^C^
Sides of CMLNs (*n* = 36)	L/R	15/21 (42/58) ^D^
Level of CMLNs (*n* = 36)	I	0 (0) ^D^
	II	6 (17) ^D^
	III	12 (33) ^D^
	IV	10 (28) ^D^
	V	1 (3) ^D^
	VI	7 (19) ^D^
Largest diameter of CMLNs (*n* = 36) (mm)		14.0 (8.0, 22.5) ^E^
Initial volume of nodule (*n* = 36) (mm^3^)		523 (148, 2010) ^E^

*M* male, *F* female, *PTC* papillary thyroid carcinoma, *FTC* follicular thyroid carcinoma, *P* positive, *N* negative, *L* left, *R* right

^A^ Number of patients

^B^ Mean ± standard deviation, with range in brackets

^C^ Number of patients, with percentage in brackets

^D^ Number of CMLNs, with percentage in brackets

^E^ Median, with P25 and P75 in brackets

### Equipment

A Siemens Acuson Sequoia 512 Ultrasound System (Siemens, Mountain View, CA, USA) with a 6 L3 linear array transducer was used to guide core needle biopsy (CNB) and ^125^I-seed implantation. A Siemens Acuson Sequoia 512 ultrasound system with a 15L8W linear array transducer or a Philips iU22 Ultrasound System (Philips Healthcare, Bothell, WA) with anL12–5 linear array transducer or a Mindray M9 Ultrasound System (Mindray, Shenzhen, China) with an L12–4 linear array transducer was used for image collection before implantation, as well as during follow-up. ^125^I seeds (radioactivity: 0.4 mCi, average energy: 27–35 keV, half-life: 60.1 days, antitumor activity: 2.0 cm) in this study were provided by Shanghai Xinke Pharmaceutical Co., Ltd.

### Pre-implantation assessment

Before ^125^I seed implantation, all CMLNs were evaluated using conventional US combined with contrast-enhanced ultrasound (CEUS). For each CMLN, three orthogonal diameters (the largest diameter and two perpendicular diameters) were measured using US. Volume was calculated using the eq. V = πabc/6 (where V represents volume, a the largest diameter, and b and c the other two perpendicular diameters). CEUS provided visualization of the blood supply region and enhancement pattern of the lesion. The US contrast agent used was Sulphur hexafluoride (SonoVueR, Bracco. International, Milan, Italy). Bolus injection of SonoVue (2.4 ml) through the elbow vein was performed for a single CEUS and each imaging acquisition lasted for at least 3 min. Thereafter, the situated level, adjacent structures, volume and flow perfusion of each CMLN were recorded in detail. The thyroglobulin (Tg) test and routine blood analysis were also performed before implantation. Tg tests were performed at the Endocrinology Laboratory, First Medical Center, PLA General Hospital. Routine blood analysis was performed at the Laboratory Department, First Medical Center, PLA General Hospital.

### Implantation procedure

The procedure was performed by an experienced US physician with more than 20 years of experience in interventional US. Patients were placed in the supine position with their neck extended. Local infiltration anesthesia with 1% lidocaine was injected at the subcutaneous puncture site and at the periphery of the lesion. The number and distribution of ^125^I seeds to be implanted were determined according to the volume and location of each CMLN. An interstitial needle (18 gauge) was gradually inserted into the lesion, and the seeds were implanted using a turntable implantation gun guided by US. Each seed was placed at intervals ranging 0.5–1.0 cm. According to the principles of the Paris system, the distribution of seeds should be arranged in a straight line and parallel to each other. The seeds should be approximately ranging 0.3 cm–0.5 cm away from the edge of the lesion and at least 1 cm away from cervical vital structures such as vessels, esophagus, trachea, and recurrent laryngeal nerves. According to the Halarism’s experienced formula: total activity (A) mCi = Da × 5, where Da denotes the mean sum value of length (L), width (W) and height (H) of the targeted lesion as (L + W + H) /3 (unit is cm). The number of seeds to be implanted was obtained using the equation: total activity (A) ÷ the average activity of a single ^125^I seed = the number of seeds required [20, 21]. Postoperative observations were conducted for 2 h. We focused on possible complications such as bleeding and hematoma during or immediately after implantation.

### Follow-up

Follow-up comprised US, CEUS, routine hematology, and thyroglobulin (Tg) levels at 1–3, 6, 9 and 12 months and every 6 months thereafter. If Tg levels were significantly elevated, for instance doubled, additional systemic examination was performed to determine whether there was distant metastasis.

The volume of each CMLN was evaluated by US, and we calculated the volume reduction rate (VRR) during follow-up as VRR = ([initial volume – final volume] × 100)/initial volume [22]. Blood perfusion of the CMLN was evaluated using CEUS. Variations in the enhancement pattern of the nodule before and after implantation were monitored. Hyperenhancement before implantation and non-enhancement or hypo-enhancement during follow-up indicated that treatments were effective [23, 24]. For those with no changes in CEUS before and after implantation, VRR should be combined with serum Tg values for evaluation. There were the following situations: (a) if VRR ≥ 50%, the treatment was deemed effective. (b). If VRR < 50%, but the serum Tg level reduction was more than 50% compared with pre-implantation, “wait and watch” would be recommended. (c). If VRR and the serum Tg level reduction were both less than 50%, further US-guided CNB should be performed to determine whether there was an active lesion residue. (d). The volume of nodules increased from the original during follow-up, indicating treatment failure. Common symptoms and severity of adverse radiation events were classified according to the criteria of Radiation Therapy Oncology Group/European Organization for Research and Treatment of Cancer (RTOG/EORTC).

### Statistical analysis

Statistical analysis was performed using SPSS statistical software (Version 23.0). Continuous data following normal distribution were represented as ^−^x ± s (range), and if not, as median (P25, P75). The Wilcoxon signed-rank test was performed to compare the changes in nodule volume before implantation and at each follow-up point as well as the serum Tg levels before implantation and at the last follow-up visit. *P* < 0.05, indicating that the difference was statistically significant.

## Results

**Fig. 1:**
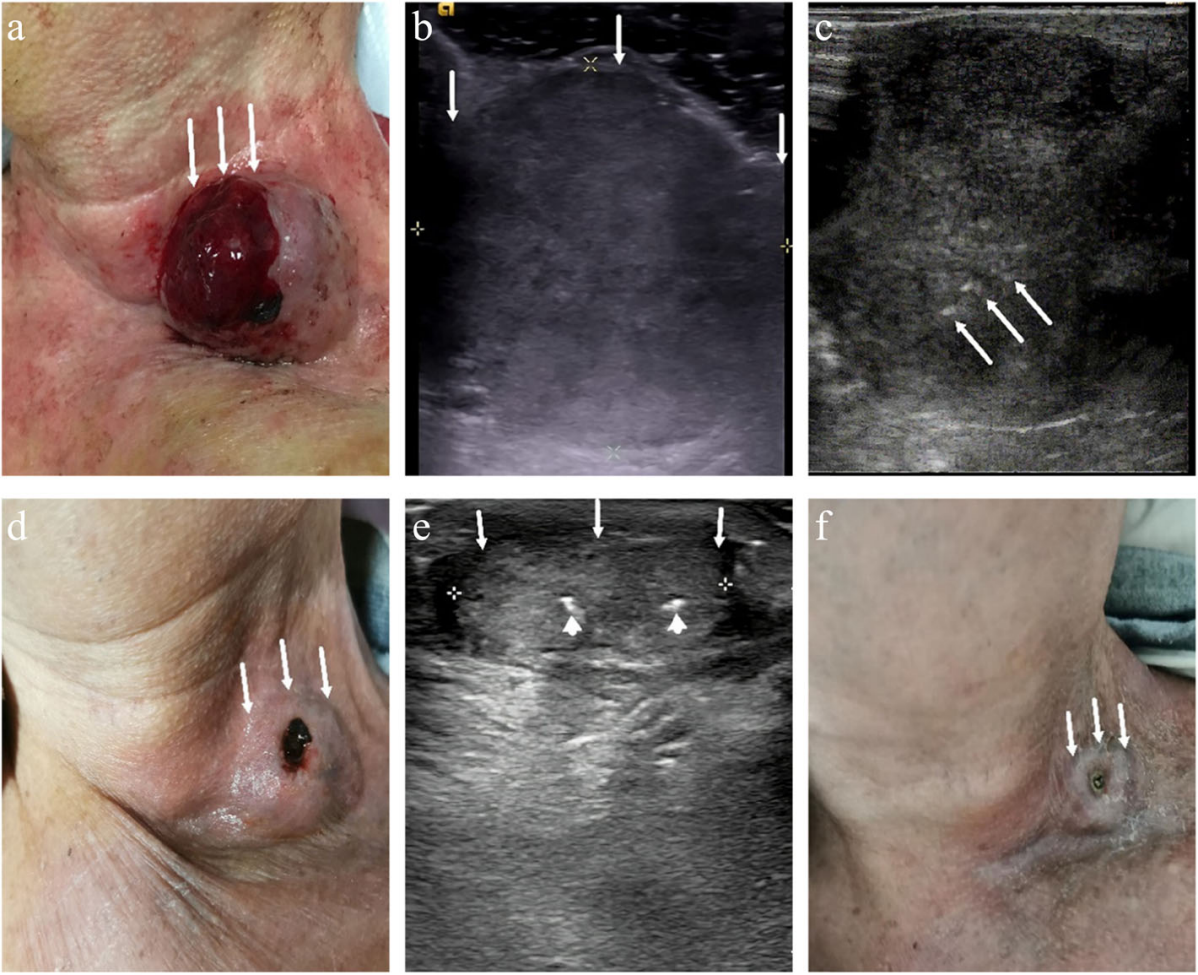
Images of ^125^I implantation in the treatment of a metastatic nodule on the left cervical level IV in an 83-year-old man who had previously undergone left lobe and isthmus excision and left neck dissection owing to papillary thyroid carcinoma. **a** Externally, the CMLN was superficial and, broad at the base, with high skin tension and bleeding apical ulcer (white arrows). **b** Conventional US image showing a hypoechoic CMLN with no lymphatic hilus was 38 mm × 37 mm × 34 mm in size and 25,017 mm^3^ in volume (white arrows). **c** During implantation, US monitoring showed that hyperechoic ^125^I seeds were implanted into the lesion (white arrows). **d** Six months after implantation, the lesion had visibly shrunk (white arrows). **e** 12 months after implantation, US showed that the lesion was 23 mm × 17 mm × 11 mm in size and 2251 mm^3^ in volume with VRR = 91% (white arrows), ^125^I seeds in the lesion (arrowheads). **f** Lesion had significantly shrunk and the apical ulcer had healed (white arrows)

**Fig. 2:**
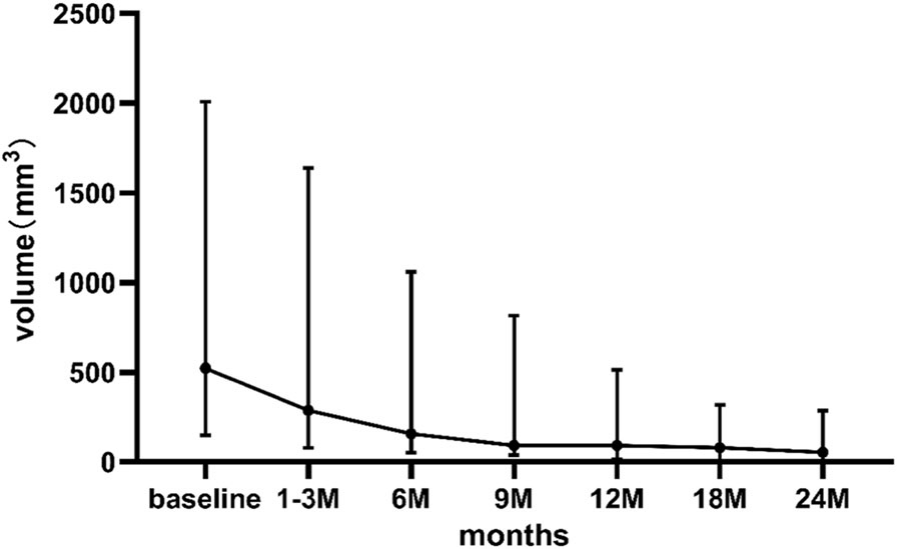
The changes of volume after implantation at each follow-up point (The dots represent the median. The two ends of bars represent P25 and P75, respectively)

**Fig. 3:**
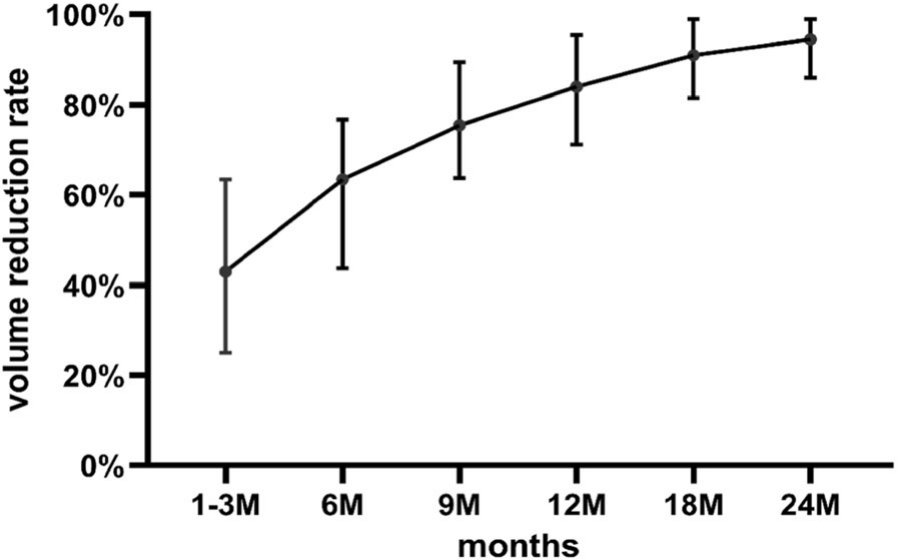
The changes of reduction ratio after implantation at each follow-up point (The dots represent the median. The two ends of bars represent P25 and P75, respectively)

As was initially planned, 36 CMLNs in 18 patients were successfully implanted with ^125^I seeds. All of them tolerated the procedure well. A total of 237 ^125^I seeds were implanted, with the least being 1 and the most being 22 seeds, and with a median of 4 (2, 11). All implantations were performed in the outpatient department. Six patients experienced mild pain after implantation and they alleviated themselves without treatment. No serious complications such as massive hemorrhage, soft tissue necrosis, neuropathy or carotid damage were noted. Furthermore, no RTOG/EORTC grade > 2 complications were observed. All patients were followed up for at least 24 months with the longest follow-up period being 50 months. Post-implantation US showed that all successfully treated nodules were reduced from their original volumes during follow-up. Before implantation, the median volumes of the CMLNs were 523 mm^3^ (148, 2010mm^3^), which decreased to 288mm^3^ (77, 1638mm^3^), 156mm^3^ (51, 1058mm^3^), 91mm^3^ (37, 816mm^3^), 91mm^3^ (14, 514mm^3^), 79mm^3^ (0, 317mm^3^) and 53mm^3^ (0, 286mm^3^) at 1–3, 6, 9, 12, 18 and 24 months after implantation, respectively. There were significant differences in nodule volume between every two follow-up visits (*P* < 0.01). The treatment outcome of 36 nodules is detailed displayed in Table 2. The median volume and median VRR of the nodules after implantation at each follow-up point are listed in Table 3. Representative findings at ^125^I-seeds implantation and at the follow-up of a CMLN case are shown in Fig. 1. The changes in median volume and VRR at each follow-up point after implantation are shown in Figs. 2 and 3, respectively. During the follow-up period (the range was 24–50 months), 25 (69%) nodules had VRR greater than 90%, of which 12(33%) nodules had VVR ≈ 100% with unclear structures and only ^125^I seed images were visible in the US. Pre-implantation, CEUS exhibited 29 (81%) lesions with hyper-enhancement, 7(19%) lesions with hypo-enhancement, whereas during the last examination, 34 (94%) lesions had hypo-enhancement or non-enhancement and, 2 (6%) lesions had hyper-enhancement. Among these two cases with persistent hyper-enhancement on CEUS, one case had a VRR of 72%, thus, continued observation was recommended, whereas the other case had a VRR of less than 50%. However, the subsequent US-guided puncture biopsy indicated that there was a small amount of lymphatic tissue in the puncture, but no definite metastatic cancer was found. During the last follow-up visit, the serum Tg level decreased from 57.0 (8.6, 114.8) ng/ml to 4.9 (0.7, 50.3) ng/ml (*P* < 0.01).

**Table 2:** Treatment outcome of the full group of 36 nodules

		Volume (mm^3^)		Tg (ng/ml)		*VRR (%)*
Patient no.	Nodule no.	Initial	24 months later	Initial	24 months later	24 months later
1	1^F^	11,448	86	>300	147.0	99
2	2	259	0	6.2	4.9	100
	3	919	0	6.2	4.9	100
	4	1507	75	6.2	4.9	95
3	5	528	60	7.9	2.9	89
	6	528	46	7.9	2.9	93
4	7	308	0	35.2	13.2	100
5	8	251	25	72.4	6.9	90
	9	55	0	72.4	6.9	100
	10	31	0	72.4	6.9	100
	11	141	0	72.4	6.9	100
6	12	299	31	57.0	1.1	90
	13	147	21	57.0	1.1	86
	14	88	0	57.0	1.1	100
7	15	4875	293	114.8	0.7	94
	16	1879	264	114.8	0.7	85
	17	2327	205	114.8	0.7	91
	18	94	0	114.8	0.7	100
8	19	4767	121	1.0	< 0.2	97
9	20	11	0	17.5	< 0.2	100
	21	15	0	17.5	< 0.2	100
10	22^G^	2010	1099	8.6	< 0.2	45
	23	513	377	8.6	< 0.2	26
11	24	13	0	< 0.2	< 0.2	100
12	25	1717	502	51	20.1	71
13	26^H^	19,225	301	119.0	60.4	98
	27	2010	63	119.0	60.4	97
	28	612	91	119.0	60.4	86
	29	518	21	119.0	60.4	96
14	30	1281	403	21.6	6.5	69
15	31	25,017	2213	>300	90.5	91
16	32	314	0	18.2	3.2	100
17	33	452	27	0.8	0.3	94
18	34	5256	1495	>300	>300	72
	35	3843	1143	>300	>300	70
	36^I^	151	94	>300	>300	38

^F^ During the 9th month of follow-up, CEUS indicated partial re-hyper-enhancement of No. 1 nodule, so the secondary implantation was performed

^G^ US-guided puncture biopsy of No.22 nodule during follow-up indicated that there was no definite metastatic cancer was found

^H^ During the third month of follow-up, VRR and the serum Tg level reduction of No. 26 nodule were both less than 50%, so the secondary implantation was performed

^I^ Although VRR reduction of No. 36 nodules were less than 50%, and the serum Tg value > 300 ng/ml, CEUS indicated low to no enhancement of the nodule

**Table 3:** The median volume and volume reduction rate of the nodules after implantation

Time	Volume (mm^3^)		Volume reduction rate (%)	
	Median volume (p25, p75)	*p*	Median VRR (p25, p75)	*p*
Before implantation	523 (148, 2010)		–	–
1-3 month later	288 (77,1638)	< 0.01	43 (25, 64)	< 0.01
6 months later	156 (51,1058)	< 0.01	64 (44, 77)	< 0.01
9 months later	91 (37,816)	< 0.01	76 (64, 90)	< 0.01
12 months later	91 (14,514)	< 0.01	84 (71, 96)	< 0.01
18 months later	79 (0,317)	< 0.01	91 (82, 100)	< 0.01
24 months later	53 (0,286)	< 0.01	95 (86, 100)	< 0.01

Throughout the follow-up period, physical examination revealed that three patients developed new CMLNs, one of which with axillary lymph node metastasis and one of which with thoracic vertebral metastasis, but there was no evidence of recurrence at the treatment sites.

## Discussion

Locoregional recurrence and metastasis may occur in 20–30% of DTC patients within 10 years after initial treatment, which is associated with an increased mortality rate [25] . Most patients diagnosed with recurrent DTC can still achieve not a poor prognosis after undergoing salvage treatment, which is based on radioactive iodine (RAI) and associated with further surgery and hormone suppression [9, 26]. Nevertheless, a small proportion of patients may develop local metastasis disease, which is not only unsuitable for repeated neck surgery but also refractory to RAI ablation. Treatment of refractory recurrences and metastases is a major challenge in the management of DTC. Few treatment options are available. Systemic therapy with TKIs, oral antiangiogenic MTKIs, has demonstrated a progression-free survival benefit based on the results of phase 3 trials [11, 27]. However, these drugs have significant toxicities that can impair the quality of life and some individuals do not tolerate them [28, 29]. External beam radiotherapy may be a modality for patients with unresectable tumors, but its role in DTC is debatable because it achieves optimized locoregional control will cause severe damage to the normal tissues and/or their functions [30]. In recent years, minimally invasive therapy has played an important role in local inoperable metastatic foci in DTC, mainly including US-guided percutaneous intervention approaches such as ethanol injection, laser ablation, radiofrequency ablation, and microwave ablation [31–33]. Each of these has feasibility and limitations. Ethanol injection is generally recommended for lesions with a maximum diameter > 10 mm, and it requires repeated treatment [34, 35]. Laser ablation tends to treat lesions with a maximum diameter of less than 10 mm [36, 37]. Radiofrequency ablation and microwave ablation have certain requirements on lesion sites owing to heat conduction and thermal damage during operation [32, 33]. Interstitial permanent ^125^I seed implantation, as a highly conformal radiotherapy modality that, delivers higher radiation doses to target areas and even to tumors closely surrounded by vital structures while sparing surrounding normal tissues, is widely applicable, almost free from the limitation of lesion location and volume, and it has been used in the treatment of unresectable malignant tumors of various organs [38–40]. It is particularly suitable for the treatment of malignant tumors of the head and neck owing to its advantages of low energy, sustained accumulated radiation, and homogenous dose distribution in the target area [17, 41]. However, there are few reports pertaining to the effects of this method in RR-DTC, and its feasibility and safety require clinical data validation.

In this study, US-guided ^125^I seed implantation was performed on 36 CMLNs of 18 DTC patients who met the inclusion criteria after thyroidectomy, and long-term follow-up was conducted to evaluate the efficacy and safety of this procedure. The somatic BRAF^V600E^ mutation, which was significantly associated with lymph node or disease metastasis and cancer-related mortality [19, 42–44], was found in 17 (94%) of all subjects. Therefore, a more aggressive approach than “watch and wait” should be adopted to control neck recurrence and metastasis in these patients. In this study, cases implanted with ^125^I seeds had a large age span (ranging 27–87), a large lesion volume span (ranging10.9–25,017.4 mm^3^), and various lesion locations. However no procedure failed, indicating that this method is widely applicable and well tolerated. All cases were successfully implanted as planned, with no technical failure, indicating that this method was well operable and could be popularized by experienced US physicians. Considering the inert biological characteristics of DTC as well as CMLNs adjacent to the trachea, vessels, nerves and other cervical critical tissues, this study adopted low-dose ^125^I brachytherapy, even so, during the last follow-up, US indicated that all treated lesions had shrunk. Significant differences in volume were found between every two follow-up visits, indicating reliable efficacy. No serious complications occurred immediately after implantation or during long-term follow-up indicating that this method is safe. In addition, the overall cost of ^125^I implantation is low, particularly for nodules with small volumes, which can reduce the economic burden on patients.

However, some limitations of this study should be considered. First, only 18 patients with 36 nodules were included in the study. Second, the volume measured by US alone was used as the basis for calculating the number of seeds to be implanted and for evaluating postoperative efficacy; if other image methods could be combined, the radiation dose calculation and efficacy evaluation could be more accurate. Third, during follow-up, two nodules underwent secondary implantation; on one hand, it demonstrated that this method had good repeatability; on the other hand, it demonstrated that there was still a radiation cold zone and the tumor had not been completely inactivated in initial treatment. Radioactive cold zones may be associated with insufficient doses. One of the above two nodules that required re-implantation is located behind the internal jugular vein and the common carotid artery, and its margin is adjacent to the vessel wall. The other is adjacent to the esophageal wall. To ensure the safety, the actual number of implanted seeds in the lesions were a little bit less than the calculated number of seeds. The association between radiation dose and safety needs to be further studied.

There are still some points that deserve consideration in this study: the subjects enrolled in the study were all diagnosed with RR-DTC; the BRAF^V600E^ gene mutation occurred in 17 out of 18 patients; however, responses to low-dose ^125^I radiotherapy were varied. After a single treatment, 25 (69%) nodules had VRRs greater than 90%, whereas 3 (8%) nodules had VRRs less than 50%, and 2 (6%) nodules required secondary implantation. In 15 (83%) patients, the disease did not progress after CMLNs were controlled, whereas in the remaining 3 (17%) patients, new metastatic lesions had developed. What accounts for these varied responses? Further large-sample studies need to be conducted.

In conclusion, US-guided ^125^I seed implantation is easy to operate, feasible, efficacious, and safe in locoregional control of RR-DTC, which can be an effective supplement for the comprehensive treatment of thyroid cancer.

## Data Availability

The datasets in this retrospective study are available from corresponding author on reasonable request. The confidential patient data should not be shared.

## Abbreviations

RR-DTC: Radioiodine refractory differentiated thyroid carcinoma
CMLN: Cervical metastatic lymph node
VRR: Volume reduction rate
CEUS: Contrast-enhanced ultrasound
